# Oral squamous cell carcinoma (OSCC) tumors from heavy alcohol consumers are associated with higher levels of TLR9 and a particular immunophenotype: Impact on patient survival

**DOI:** 10.3389/fimmu.2022.941667

**Published:** 2022-08-05

**Authors:** Nicolás Bolesina, Gerardo Gatti, Silvia López de Blanc, Sabrina Dhooge, Darío Rocha, Elmer Fernandez, Ruth Ferreyra, Vanesa Palla, Verónica Grupe, Rosana Morelatto, Mariana Maccioni

**Affiliations:** ^1^ Departamento de Patología Oral, Cátedra de Estomatología, Facultad de Odontología, Universidad Nacional de Córdoba, Córdoba, Argentina; ^2^ Fundación para el Progreso de la Medicina. Laboratorio de Investigación en Cáncer, Córdoba, Argentina; ^3^ Centro de Investigación y Desarrollo en Inmunología y Enfermedades Infecciosas, CIDIE-CONICET, Universidad Católica de Córdoba; Facultad de Ciencias Exactas, Físicas y Naturales, Universidad Nacional de Córdoba, Córdoba, Argentina; ^4^ Centro de Investigaciones en Bioquímica Clínica e Inmunología, CIBICI-CONICET, Departamento de Bioquímica Clínica, Facultad de Ciencias Químicas, Universidad Nacional de Córdoba, Córdoba, Argentina

**Keywords:** oral squamous cell carcinoma, immunophenotype, alcohol consumption, CD8+T cell infiltration, TLR9

## Abstract

Oral squamous cell carcinoma (OSCC) is one of the most frequent types of oral cancer in developing countries and its burden correlates with exposure to tobacco and excessive alcohol consumption. Toll like receptors (TLRs) are major sensors of inflammatory stimuli, from both microbial and sterile causes and as such, they have been related to tumor progression and metastasis. Here, we evaluated the expression of TLR2, 4 and 9 as well as CD3+, CD8+ and Granzyme B+ cell infiltration by immunohistochemistry in oral samples of 30 patients with OSCC, classified according to their consumption of alcohol. Our findings indicate that there is a significant association between heavy alcohol consumption and tumors with higher expression levels of TLR9. Moreover, patients with TLR9high tumors, as well as those who indicated high consumption of alcohol exhibited a diminished overall survival. TCGA data analysis indicated that TLR9high tumors express a significant increase in some genes related with the oral cavity itself, inflammation and tumor promotion. Our analysis of tumor infiltrating leukocytes demonstrated that the major differences perceived in heavy alcohol consumers was the location of CD8+ T cells infiltrating the tumor, which showed lower numbers intratumorally. Our data suggest the existence of a pathogenic loop that involves alcohol consumption, high TLR9 expression and the immunophenotype, which might have a profound impact on the progression of the disease.

## Introduction

Head and neck cancer was identified as the seventh most common cancer worldwide in 2018, with 890,000 new cases and 450,000 deaths being reported (1). Oral squamous cell carcinoma (OSCC) is the most prevalent subgroup of head and neck cancer and includes the lips, the tongue, the gums, the buccal mucosa, the floor of the mouth, the hard palate, and the alveolar ridge. It represents a major cause of morbidity and mortality worldwide, especially in Southern Asia and the Pacific islands (2). It arises as a consequence of multifactorial risk factors that include tobacco and alcohol, chronic inflammation, ultraviolet radiation (for lip cancer), human papilloma virus (HPV) or Candida infections, immunosuppression, genetic predisposition, and diet. However, tobacco smoking and alcohol consumption are considered as the main causal factors (3, 4). OSCC is still a leading cause of cancer death among men in many places and despite several advancements in therapeutic approaches, the 5-year survival rate is around 50% (1).

Toll-like receptors are an evolutionary conserved family of transmembrane receptors, key players in the initiation of the immune response. They are present almost in any cell type and tissue in the body, although they are preferentially expressed on antigen presenting cells. Twelve members of the TLR family have been identified in mammals so far (5, 6) and it is well established that, besides interacting with microbial molecular structures to trigger the inflammatory response, they also recognize self-molecules that are released, secreted or exposed by cells that are suffering stress or damage. These self-molecules are called “danger associated molecular patterns” or “DAMPs”. Thus, they are at the crossroad between immunity and inflammation, being important components of immunotherapeutic strategies designed to awaken the immune response against tumor antigens, but also mediating chronic inflammatory processes involved in carcinogenesis and promotion of the neoplastic lesion (7).

Up to 10^10^ bacteria reside in the oral cavity, which in physiologic conditions presents a relatively small number of resident immune cells, including neutrophils, lymphocytes, and monocytes/macrophages (5). As in other mucosal sites, the expression of TLRs in oral epithelial cells seems to be tightly controlled as a way of maintaining tissue homeostasis in a site full of commensal microflora. Most TLRs are expressed in the oral epithelial cells, at mRNA and protein levels, but their levels are modified and their location can change from basal to superficial layers when invading pathogens are detected (8–10). Except for TLR7 and TLR8, which do not increase their protein levels in inflammatory conditions, TLR1, TLR3, TLR4, TLR5, and TLR9 expression depends on the state of the tissue (inflamed vs. non-inflamed) (5, 11).

The expression of TLRs has been thoroughly investigated in most types of cancer and oral cancer is not an exception. In general, the levels of the most studied TLRs (TLR9, TLR4, TLR2) are enhanced in OSCC tissues compared to healthy controls and in most cases they have been associated with invasion and metastasis (11). Usually, most studies have relied on measurements of mRNA levels, immunohistochemistry and correlation analysis with clinical data such as tumor size and presence or not of metastasis and invasion. However, there are few studies trying to associate their enhanced expression with the presence of carcinogenic stimuli. In this study we have analyzed the expression levels of TLR9, TLR4 and TLR2 in OSCC samples by immunohistochemistry and found that patients with excessive alcohol consumption exhibit a higher frequency of TLR9^high^ tumors. Also, these patients have a reduced survival. We have characterized the differences of immune infiltrate in tumors from heavy and low alcohol consumers and distinct TLR9 expression levels (TLR9^high^ and ^low^). We also evaluated the differentially expressed genes by interrogating TCGA data. Genes like ADAM6 (a member of the family of A disintegrin and metalloproteases) and TNFRSF13B (member of the superfamily of TNF receptors), which contribute to the inflammatory process and have been involved in cell migration and metastasis, are significantly elevated in TLR9^high^ tumors. Our data suggest the existence of a pathogenic loop that involves alcohol consumption, high TLR9 expression, and the immunophenotype, which could have a profound impact on the progression of the disease.

## Material and methods

### Study population and tissue source

This is a retrospective, observational and analytical study that included 30 biopsy samples from patients with a pathological diagnosis of OSCC as a primary tumor, with complete clinical history and HPV determination as shown in **Table 1**. All the patients attended at the Stomatology Service of the Faculty of Dentistry of the National University of Cordoba, Argentina. Patients who had consumed anti-inflammatory drugs or had been treated with chemo and/or radiotherapy were excluded. Written informed consent was obtained from all subjects and all studies were conducted in accordance with Good Clinical Practice guidelines and the Declaration of Helsinki. The protocol was approved by the Academic Committee for Health Research of the Facultad de Odontologia. Universidad Nacional de Córdoba, (UNC) (Project 31, 06/25/2015).

**Table 1:** Patient characteristics.

Patient characteristics	n (%)
Age (years)	
>60	18 (60)
<60	12 (40)
Gender	
Female	15 (50)
Male	15 (50)
Tumor differentiation	
Well	11 (39)
Moderate/poorly	18 (61)
Cancer stage	
1/11 (initial)	19 (68)
III/IV (advanced)	9 (32)
Smoking habit	
High	15 (50)
Low	15 (50)
Alcohol consumption	
High	9 (30)
Low	21 (70)
HPV	
Yes	12 (41)
No	17 (59)

Tumors were staged according to the UICC-TNM classification of malignant tumors (12). Stages I and II were defined as initial and stages III and IV as advanced. Also, tumor differentiation was defined as well or moderate/poorly according to the World Health Organization (13).

Cumulative tobacco use was evaluated following the criteria established by Biondi et al., 1998 (14). A subject who smoked more than 200,000 cigarettes during his lifespan was considered a high consumer. In the same way, alcohol consumption was registered taking into account the type and quantity of alcoholic beverages consumed. One unit of alcohol per day (one drink) was considered as regular alcohol consumption, according to Pentenero et al., 2011 (15). The consumption of at least one alcohol unit per day (1 unit=8–10g of ethanol=1 glass of wine=1/4l of beer=1 measure of liqueur) was considered alcohol exposure.

Patients consuming more than 60g of alcohol/day were considered heavy alcohol consumers, according to the Ministry of Health of Spain (16) and the National Institute of Alcohol Abuse and Alcoholism of the U.S (17).

Other clinical aspects such as HPV determination were evaluated following protocols established. Briefly, DNA-HPV was detected *via* PCR using Bioneer AccuPrep genomic DNA Extraction kit, and consensus primers MY09 and MY11.

### Immunohistochemistry

All collected tissue samples were fixed in formalin, embedded in paraffin and cut at 4µm and then standard IHC staining was performed.

For detection of the TLR receptors, the following antibodies were used: anti-TLR2 (H-175, rabbit polyclonal antibody against amino acids 180 to 354 of TLR2 of human origin, Santa Cruz Biotechnology); anti-TLR4 (H-80, rabbit polyclonal antibody against amino acids 242-321 of TLR4 of human origin, Santa Cruz Biotechnology) and anti-TLR9 (H-100, rabbit polyclonal IgG antibody against TLR9 of human origin, Santa Cruz Biotechnology).

**Figure 1:**
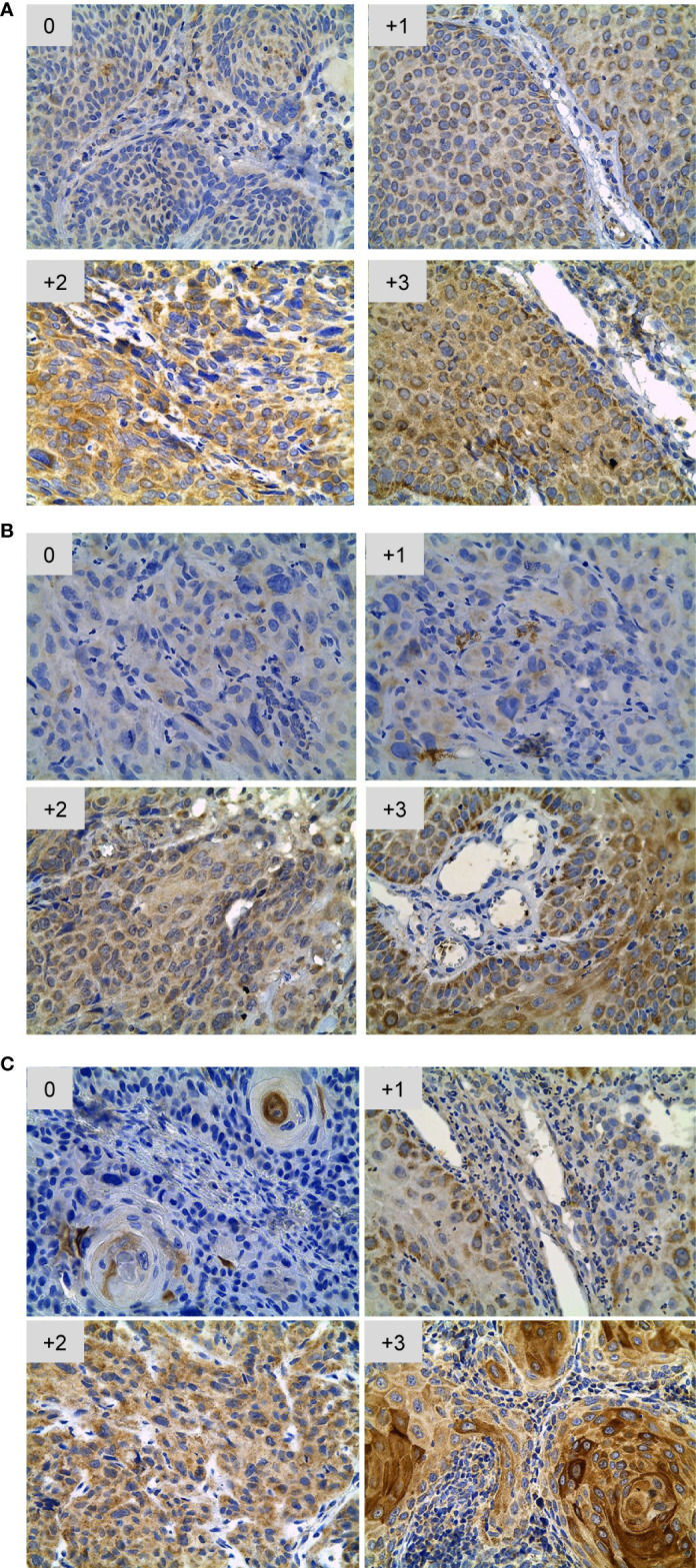
Scoring system for immunohistochemical staining of TLR2 **(A)**, TLR4 **(B)** and TLR9 **(C)** in OSCC. Each panel shows representative pictures of the increasing scores (from 0 to +3) according to the different expression levels of TLR expression. The intensity of the staining is designated as 0 when no tumor cells are stained; 1+ when 10- 20% of cells are stained (weak), 2+ when 20- 50% of cells are stained (moderate), and 3+ when >50% of cells are stained (strong). Micrographs were obtained at 40x.

TLR staining patterns were evaluated and scored based on intensity and percentage of positive cells as previously described (18, 19). The score is designated as 0 when no tumor cells stain, 1+ when 10–20% of cells stain (weak), 2+ when 20–50% of cells stain (moderate), and 3+ when > 50% of cells stain (strong). The immunohistochemistry scoring was performed by two observers that were blinded and the degree of agreement was good (Kappa statistics: 0.8). Tumors were then classified as TLR low (< 2 score) or TLR high (≥ 2 score). **Figure 1** shows representative images of each staining pattern for TLR2, TLR4 and TLR9.

CD3+, CD4+, CD8+ and Granzyme B+ cells were visualized at 40x of magnification and quantified using the Image J software. The results show the average number obtained after counting infiltrating cells in 3 fields per patient.

To study the location of CD3+, CD4+ and CD8+ T cell infiltration, we analyzed the number of cells/fields at the invasive margin or at the center of the tumor (intratumoral). Therefore, 3 fields per sample were visualized at 20x magnification and a scoring system was established as follows: 0 when no cells were present, 1+ when 10–20% of cells were present, 2+ when 20–50% of cells were present, and 3+ when > 50% of cells were present. Each sample received two scores corresponding to the location of the cells: one score for the invasive margin and another one for the center (intratumoral).

### TCGA data analysis and MIXTURE

The results reported here are partially based on data generated by The Cancer Genome Atlas (TCGA: https://www.cancer.gov/tcga). MIXTURE, a ν-support vector regression-based noise constrained recursive feature selection algorithm based on validated immune cell molecular signatures (20), was used to estimate tumor immune infiltration from expression data. TLR9 expression was categorized as high or low according to the median TLR9 expression value.

### Statistical analysis

Data handling, analysis, and graphic representation (all shown as mean ± SEM) were performed using Prism 8.0.2 (GraphPad Software) and R studio (https://www.rstudio.com/products/rstudio/download/). For the comparison between two groups, a difference of proportions Z-test was performed. A p< 0.05 was considered statistically significant. Survival analyses were performed in R with Kaplan-Meier estimator and log-rank test. The comparisons for the abundance of TLR9, TLR4 and TLR2 between the different stages were performed with the Student T test. The comparisons for the abundance of CD8+ T cells, CD3+ T cells and Granzyme B+ cells between TLR9^high^ and TLR9^low^ groups, was performed with the Mann- Whitney test. In the MIXTURE analysis, the Wilcoxon test was used.

## Results

### Patients with OSCC tumors expressing elevated levels of TLR9, but not of TLR2 or TLR4, exhibit a reduced 10-years overall survival

**Figure 2:**
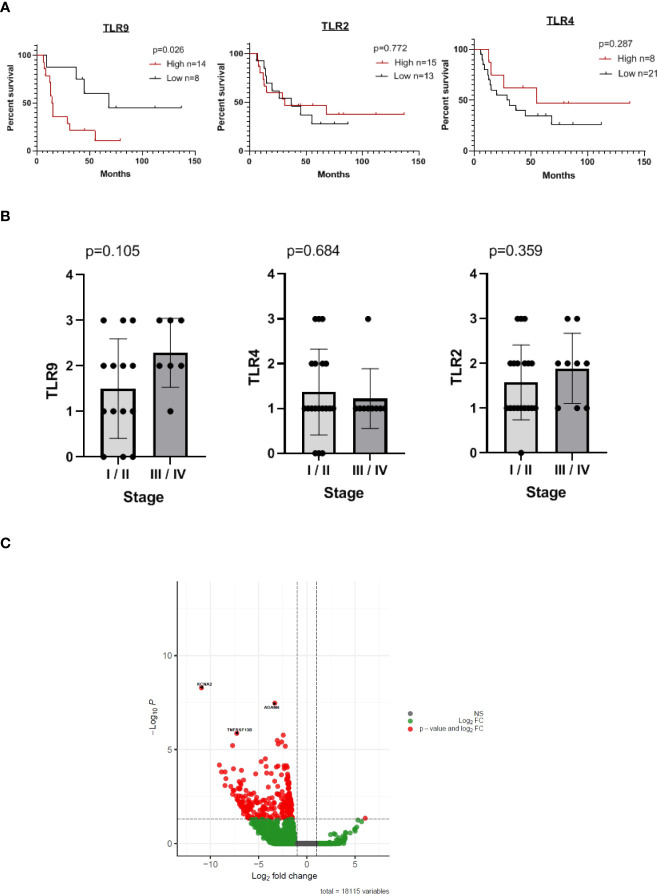
Patients with OSCC tumors expressing elevated levels of TLR9, but not of TLR2 or TLR4, exhibit a reduced 10-years overall survival. **(A)** Kaplan-Meier plots of overall survival of patients from the cohort under study. Patients were categorized as high or low TLR9, TLR4 and TLR2 as detailed in M&M. **(B)** Patients were categorized according to UICC- TNM criteria. Stages I and II were considered “initial” whereas stages III and IV were considered “advanced”. **(C)** Volcano plot showing the differential expressed genes in samples analyzed from the TCGA and categorized as TLR9^low^ and ^high^ according to their median expression value. The plot shows genes that have a significantly up-regulated expression in TLR9^high^ samples (red dots) based on both biological difference (absolute log2; estimated fold change) and statistical difference (-log10; p value). Negative log fold changes indicate that the expression is higher in the TLR9 ^high^ samples.

There exists abundant data regarding the expression of different TLRs in the oral cavity, both in physiological and in pathological conditions; however, it is not clear yet if the up-regulation observed in inflammatory settings such as OSCC has a prognostic meaning. We measured TLR9, 2 and 4 expressions in our tumor samples and classified our cohort of patients as showing high (≥2) or low (<2) score expression levels of TLR9 (n=22), TLR2 (n=28) and TLR4 (n=29) (**Figures 1**, **2A**). Of note, the staining was observed mainly in tumor cells and not in infiltrating immune leukocytes. We then analyzed the epidemiological data collected in **Table 1** to calculate the 10-year survival rates in our cohort according to these three TLRs expression levels. Interestingly, patients whose tumors showed an enhanced expression of TLR9, but not of TLR4 or TLR2, showed a significant decrease in their overall survival (**Figure 2A**). It could be argued that the pattern of survival observed could be driven by the stages of the disease and not by the TLR9 expression. However as can be seen in **Figure 2B**, there are non-significant differences between the expression levels of TLR9, 4 and 2 in tumors of different stages indicating that, in our cohort, other factors besides the conventional disease staging drives the overall survival.

We next interrogated the TCGA database to see which genes that were differentially expressed between TLR9^high^ and TLR9^low^ OSCC samples could explain the marked reduction in the overall survival observed in patients with TLR9^high^ tumors. **Figure 2C** shows the myriad of genes whose expression increases significantly (red dots) in TLR9^high^ vs TLR9^low^ tumors. Among those genes, we focused on KCNA2, TNFRSF13B and ADAM6, which exhibit a 11x, 7x and 3.5x fold increase respectively, since they were among the 9 differential expressed genes with p values <0.00001 that have a clearer biological role in cancer.

### A significant proportion of OSCC tumors from heavy alcohol consumers express high levels of TLR9

In neurodegenerative or liver diseases, the molecular and cellular mechanisms that link alcohol and the disruption of the organ homeostasis have been thoroughly investigated (21, 22). Indeed, in these studies, innate immune receptors play a protagonist role. In contrast, whereas tobacco and alcohol consumption are very well-known carcinogens in oral cancer and the altered presence of TLRs has been extensively documented, their association has been scarcely analyzed.

**Figure 3:**
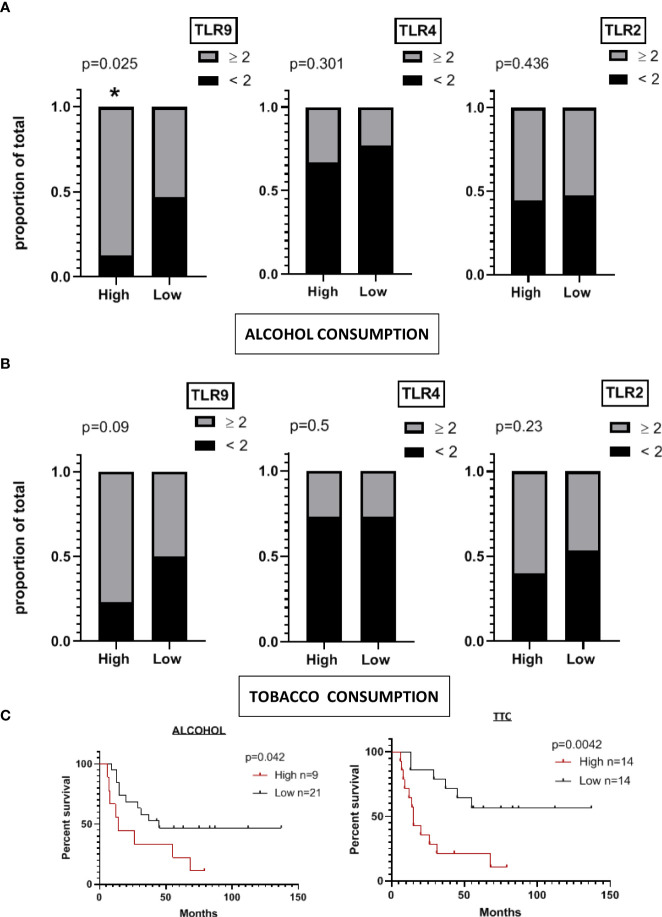
A significant proportion of OSCC tumors from heavy alcohol consumers express higher levels of TLR9. **(A)** Frequency of samples expressing scores ≥ 2 or < 2 of TLR9, TLR4 and TLR2 among patients categorized as heavy (n=9; high) or low alcohol consumers (n=21) (alcohol consumption > or <60 g/day). **(B)** Patients were categorized as high or low tobacco consumers. More than 200.000 cigarettes during his lifetime was considered to be a high consumer. A difference of proportions Z-test was performed to calculate the statistical significance. **(C)** Kaplan-Meier plots of overall survival of patients categorized as high or low alcohol or tobacco (TTC) consumers. * p < 0.05 was considered statistically significant.

To see if the expression levels of TLR9, 4 or 2 was somehow related to alcohol consumption, we divided our cohort (n=28) according to their alcohol consumption (60g/day> or < 60g/day) and analyzed the frequency of tumors expressing high or low levels of TLR9, 4 and 2 in each group (**Figure 3A**). A similar approach was done splitting the cohort according to the levels of tobacco intake in each group (**Figure 3B**). We found that heavy alcohol consumers show a significantly elevated proportion of tumors expressing higher scores of TLR9 staining, but not of TLR4 or 2, compared to those patients consuming less alcohol (**Figure 3A**). Indeed, 88% of the patients that drank more than 60g alcohol/day and were cataloged as heavy alcohol consumers exhibit tumor samples that showed an enhanced expression of TLR9. In contrast, only 50% of the lower alcohol consumers exhibited samples with high TLR9 expression (**Figure 3A**). When we looked at tobacco consumers, there was not a significant distribution of TLR^high^ expressing tumors in a particular subgroup of patients. However, we could observe a trend in associating patients with higher tobacco intake with samples with an enhanced TLR9 expression (**Figure 3B**). Interestingly, both heavy alcohol and tobacco consumers showed a significant reduction in their 10-year overall survival (**Figure 3C**).

In conclusion, these findings indicate that there is an uneven distribution of TLR9^high^ tumor samples, with a significant association between heavy alcohol consumption and tumors with higher expression levels of TLR9.

### Heavy alcohol consumers exhibit a significant reduction in intratumoral CD8+ T cells, whereas the total number of CD3+ T cells, CD8+ T cells and Granzyme B+ is not altered

**Figure 4:**
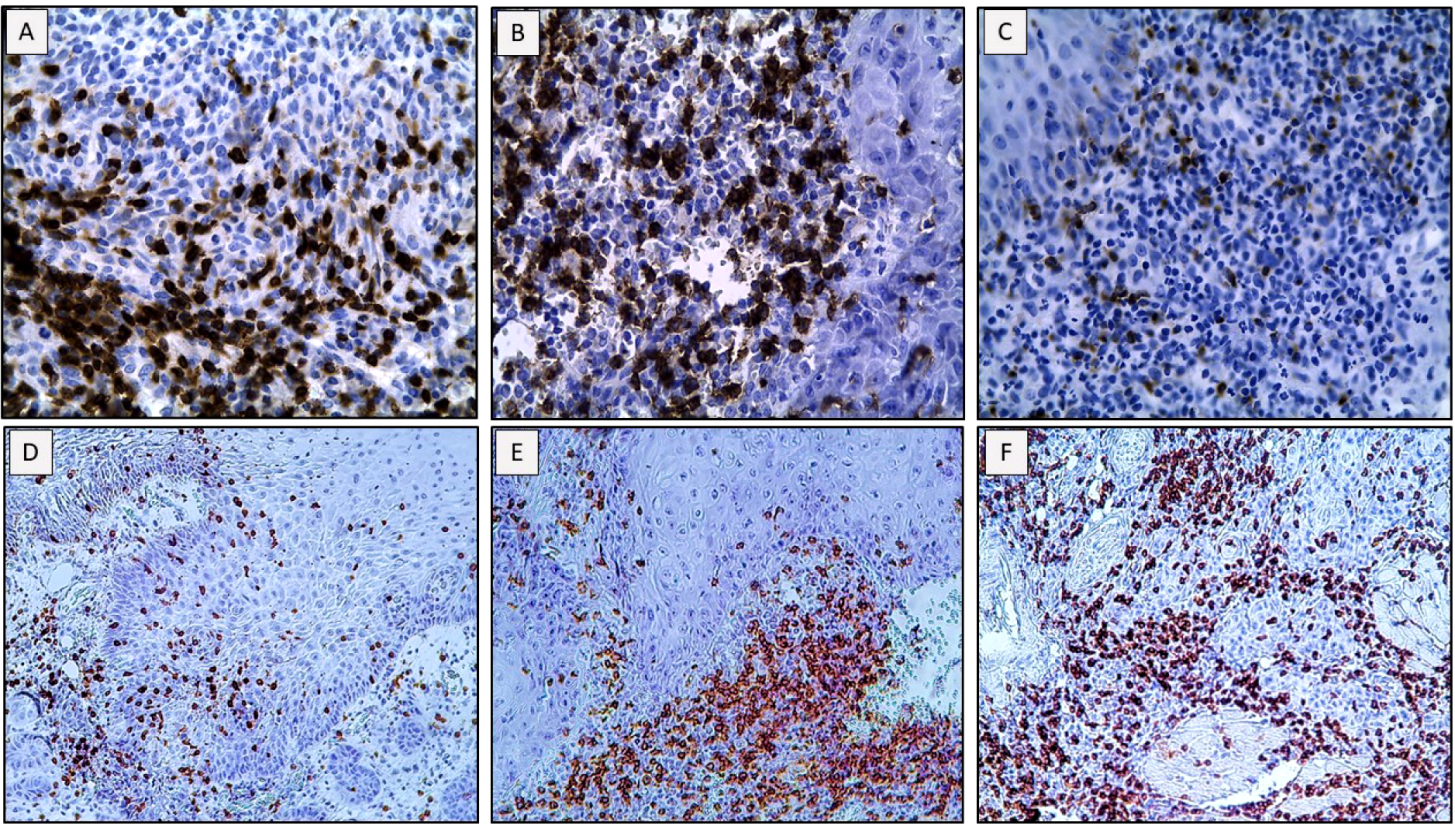
Representative pictures of immunohistochemistry images used to quantify CD3+, CD8+ and Granzyme B+ cells and to analyze their local distribution inside the tumor samples. **(A)** CD3+ cells; **(B)** CD8+ cells; **(C)** Granzyme B+ cells. Micrographs were obtained at 40x. **(D)** CD8+ cells located intratumorally in the center of the tumor or **(E)** CD8+ cells located mainly at the invasive margin; **(F)** in both the invasive margin and in the center of the tumor. Micrographs were obtained at 20x.

**Figure 5:**
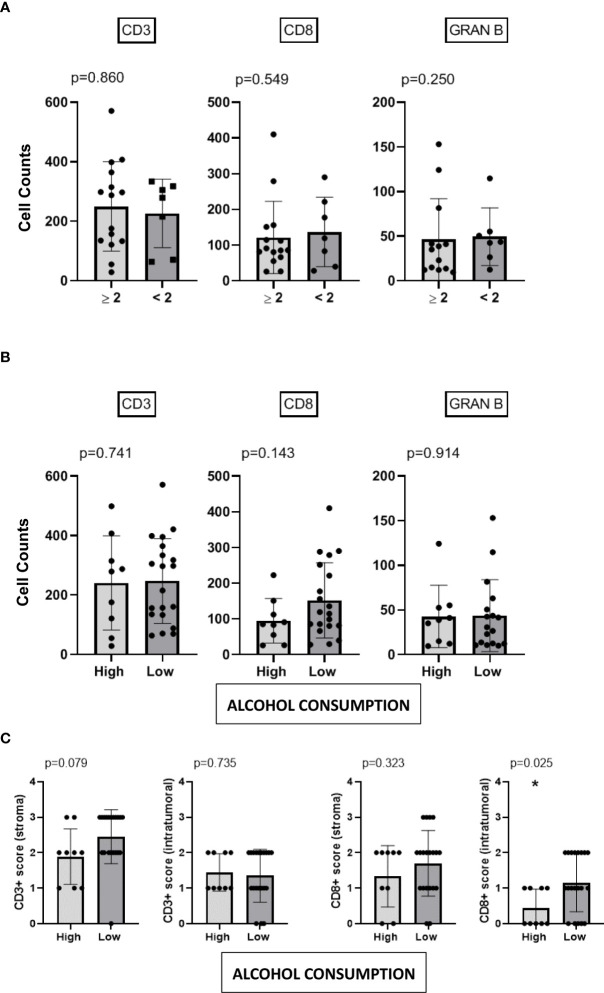
Heavy alcohol consumers exhibit a significant reduction of CD8+ T cells in the tumor center, whereas the total number of CD3+ T cells, CD8+ cells and Granzyme B+ is not altered. Total cell counts of CD3+, CD8+ and Granzyme B (GRANB+) cells (average count from 3 fields visualized at 40x) in patients categorized as ≥ 2 or < 2 of TLR9 **(A)** and high (heavy) or low alcohol consumers **(B)**. Location of CD3+ and CD8+ cells (at the invasive margin or in the center of the tumor) in the tumor samples expressed according to the scoring system detailed in M&M in patients categorized as high (heavy) or low alcohol consumers **(C)**. *p < 0.05 was considered statistically significant.

**Figure 6:**
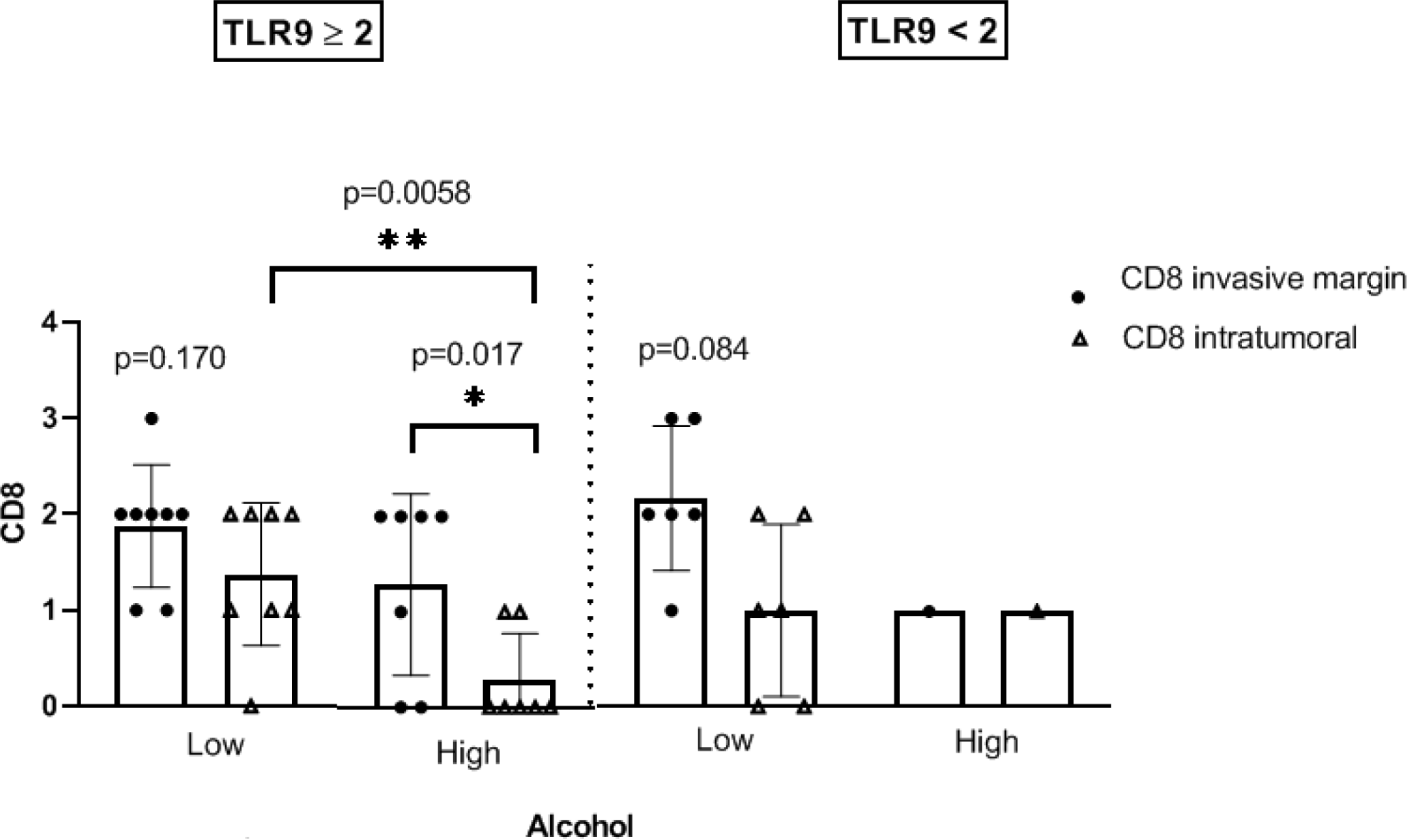
TLR9^high^ tumors that belonged to heavy alcohol consumers presented significantly fewer CD8+ T cells in the center of the tumor compared to those TLR9^high^ tumors from low alcohol consumers. Location and scores of CD8+ cells in TLR9^high^ and ^low^ samples from patients who were cataloged as high (heavy) or low alcohol consumers. Student T test was performed. *p < 0.05; **p < 0.001 were considered statistically significant.

We then quantified the total number of CD3+, CD8+ and Granzyme B+ cells and analyzed their spatial distribution (invasive margin vs intratumoral) by conventional IHC (**Figure 4**). CD4+ T cells were also analyzed but non-statistically significant differences were found among the groups (data not shown). As can be seen in **Figure 5A**, the total number of cells in these populations is not modified between tumors showing distinct scores of TLR9. Similar results were obtained when we analyzed the infiltration of immune cells in tumor samples of our cohort categorized according to their alcohol consumption levels (**Figure 5B**). However, whereas the numbers and location of CD3+ T cells do not vary significantly between patients with different levels of alcohol consumption, the location of CD8+ T cells is modified in tumor samples from heavy alcohol consumers. Indeed, they seem to develop tumors that not only express higher levels of TLR9, but also exhibit a reduced number of intratumoral CD8+ T cells (**Figure 5C**). This is clearly demonstrated in **Figure 6** that shows that in our study cohort: a) most heavy alcohol consumers display TLR9^high^ tumors (as shown previously in **Figure 3**); b) those TLR9^high^ tumors that belonged to heavy alcohol consumers presented significantly fewer CD8+ T cells in the tumor center compared to those TLR9^high^ tumors from low alcohol consumers and c) tumors from low alcohol consumers develop tumors with CD8+T cells that are distributed homogeneously between the invasive margins and in the center of the tumor, independently of TLR9 expression.

To corroborate our results analyzing data from larger cohorts, we used TCGA data to see if a particular gene signature could explain the distribution of CD8+T cells in heavy alcohol consumers with TLR9^high^ tumors, but we could not identify a precise set of genes (data not shown).

**Figure 7:**
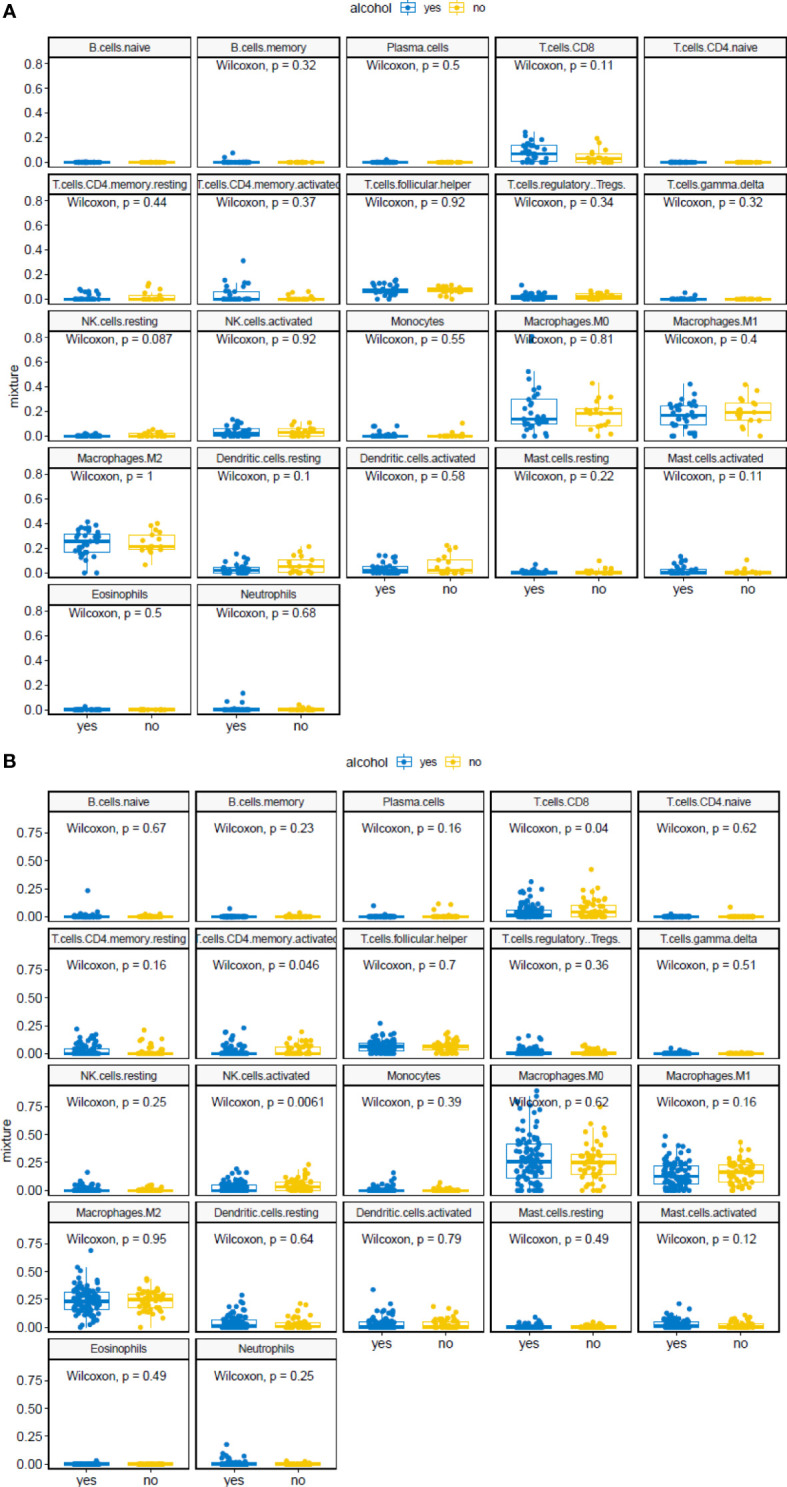
MIXTURE analysis of OSCC patients with or without alcohol consumption. Relative abundance of tumor infiltrating leukocytes in OSCC from patients with or without alcohol consumption, classified according to their tumor stages. Stages I-II **(A)** or III-IV **(B)**. Wilcoxon signed-rank test was performed.

Thus, we used TCGA data combined with MIXTURE, an immune tumor microenvironment estimation method based on gene expression data (n=200 samples). In this case, the algorithm categorizes the data into those patients who drink alcohol or not (yes or no), without specifying neither levels of consumption nor location of the infiltrating cells. Interestingly, using this algorithm, non-significant differences between these two groups of patients were seen regarding the numbers of tumor infiltrating leukocytes in initial stage tumors (**Figure 7A**). A slight increase in CD8+ T cells was observed as tumor stages progressed, which is accompanied with minor augment of activated CD4+ T cell numbers. However, the most remarkable change in the immune infiltration detected by MIXTURE is the elevated number of activated NK cells in advanced stage tumors from non-alcohol consumers (**Figure 7B**), an aspect that deserves further investigation.

In sum, our analysis of tumor infiltrating leukocytes in tumor samples of patients with different consumption habits indicate that the major differences perceived in patients with heavy alcohol intake are that their tumors express higher levels of TLR9, with decreased numbers of CD8+ cells inside the tumor center and lower numbers of activated NK cells. Remarkably, both populations, CD8+ and NK cells represent the tumor killing cells par excellence.

## Discussion

The role of TLRs in cancer have been thoroughly investigated along the last decade and evidence indicate that these receptors can act as double-edged swords, promoting chronic inflammation, tumor cell migration and invasion, but also being required for an immunogenic cell death and the success of many types of therapies (6). In this work, we have analyzed tumor samples from a cohort that included 30 patients from Argentina and investigated possible connections among the expression levels of TLR9, 4 and 2, the consumption habits of the patients and CD8+T cell infiltration by IHC, regardless of their HPV status. As a support of our findings, we relied on MIXTURE, an algorithm that can predict the type of immune infiltration in a particular tumor type by collecting gene expression data from the TCGA (20).

Surprisingly, although TLR9 expression in OSCC is very well studied, there is scarce data regarding its prognostic value (23). Indeed, it is well known that TLR9 expression is enhanced in OSCC compared to normal tissue and it has significantly been associated with tumor size, clinical stage and proliferative status. Moreover, *in vitro* stimulation of oral cell lines with a TLR9 agonist, up-regulates the secretion of molecules involved in cancer cell invasion (8, 23–26). However, in spite of the existence of many reports supporting a putative correlation of TLR9 expression with worsening clinical parameters, its impact on the survival is confusing due to small sample sizes and other inherent methodological limitations (27). Our first compelling finding is the fact that, in our cohort, patients presenting TLR9^high^ tumors, have a reduced overall survival compared to those who present TLR9^low^ samples. Non-significant differences in the survival rates of the patients were observed when the expression of TLR4 or TLR2 was studied.

In an attempt to understand the impact of TLR9 expression levels on OSCC prognosis, we interrogated the TCGA database. It is important to note that data collected from the TCGA comes from the bulk lysate and as such involves tumor stroma, immune cells, and tumor cells. However, it could provide a hint regarding which molecular processes could be involved in TLR9^high^ tumors. Interestingly, many genes showed an up-regulated expression in TLR9^high^ vs TLR9^low^ tumors, but we could not detect genes presenting a significant down regulation of their expression. Some of them, like KCNA2 (a voltage-gated K+channel, present at the fungiform papillae in the tongue and acts as the receptor to polyunsaturated fatty acid chemoreception), reflect specific features of the oral cavity (28, 29). Others, such as TNFRSF13B (member of the superfamily of TNF receptors) and ADAM6 (a member of the family of A disintegrin and metalloproteases) and contribute to a huge variety of biological processes such as cell growth, cell differentiation and metabolism and can participate in tumor promotion (30, 31).

ADAM6, for example is a member of the family of A disintegrin and metalloproteases, which are proteases involved in “ectodomain shedding” of diverse growth factors, cytokines, receptors and adhesion molecules (31–34). ADAM family of metalloproteases play a decisive role in regulating cell phenotype *via* their effects on cell adhesion, migration, proteolysis and signaling, and consequently, affecting the progression of the tumor. Another up-regulated gene is TNFRSF13B (TACI), which belongs to the superfamily of TNF receptors (TNF(R)SF) and contributes to a huge variety of biological processes such as cell growth, cell differentiation and metabolism. Whereas its role in B cell biology is very well known, it has recently been revealed other functions in solid tumor pathogenesis, such as in breast cancer (30). In sum, the fact that these genes are up-regulated are in accordance with our findings demonstrating a shorter survival in patients bearing TLR9^high^ tumors.

Another important result from our work is the significant association observed between heavy alcohol consumers and TLR9^high^ tumors. Indeed, alcohol consumption has been indicated as a risk factor for a number of cancers and its main metabolite, acetaldehyde, was declared a carcinogen by the International Agency for Research on Cancer (IARC) in 1999 and has been confirmed as a Group 1 carcinogen to humans in 2009 (35). Besides its genotoxic role, alcohol strongly disrupts the organ homeostasis. In general, alcohol is capable of: a) changing the microbiome composition, selecting microbes that are capable of high-rate acetaldehyde metabolism or are more tolerant to acetaldehyde; b) directly inducing tissue damage and consequently, increasing the release of DAMPs such as self-DNA; c) generating reactive oxygen species and lipid-originated metabolites and; d) promoting the recruitment of immune cells and thus, chronic inflammation. In all these processes, innate immune receptors have been involved. Regarding TLR9 expression and alcohol, much of what is currently known comes from experiments done in *TLR9*KO mice models of alcohol-induced liver injury, but information is still controversial. There are reports indicating that TLR9 signaling in hepatocytes counteract alcohol-induced hepatotoxicity but worsens the proinflammatory response (36, 37), while other authors indicate just the opposite (38). Therefore, it is still not clear how alcohol consumption is related to TLR9 expression and its signaling, but they both affect the outcome of the patient.

An intriguing aspect of our results is the selective location that CD8+ cells display in TLR9^high^ tumors from heavy alcohol consumers. Interestingly, this group of patients selectively show less CD8+ cells inside the tumor center (intratumoral), although no significant differences in the total numbers of infiltrating CD8+ cells was observed. The fact that TLR9^high^ tumors from low alcohol consumers do not present this selective CD8+ cell location (**Figure 6**), suggests that a conjunction of factors could be taking place in heavy alcohol consumer patients with TLR9^high^ samples. It can be hypothesized that the expression of certain chemokines is modified in TLR9^high^ tumors from heavy alcohol consumers that could be impeding the recruitment of these cells intratumorally. Another possibility would be that the activation state of these CD8+ cells were inefficient or that excessive alcohol consumption reinforces TLR9 signaling and all the protumorigenic effects involved in this pathway.

On the other hand, in our study population, there was only one patient with heavy consumption of alcohol and a TLR9^low^ tumor (12% of our cohort). Thus, a larger number of samples would be needed to see if this particular distribution of CD8+ cells is independent of TLR9 expression and only linked to alcohol consumption.

In any case, our results open up new avenues to investigate the possible interaction between consumption habits and immune characteristics of oral cancer and provide new evidence to consider TLR9 as a therapeutic target in OSCC.

## Data availability statement

The original contributions presented in the study are included in the article/supplementary material. Further inquiries can be directed to the corresponding authors.

## Ethics statement

The studies involving human participants were reviewed and approved by Academic Committee for Health Research of the Faculty of Dentistry – National University of Cordoba UNC (Project 31, 06/25/2015). The patients/participants provided their written informed consent to participate in this study.

## Author contributions

NB and GG contributed equally to the work by collecting the samples and performing the IHC analysis and infiltration characterization. SLB and RM were in charge of collecting the sample as and clinical characterization of the cohort. DR and EF performed the bioinformatic interrogation of the TCGA and MIXTURE analysis. SD did the statistical analysis of the data. RF, VP, and VG participated as pathologists and assessed tumor sample features. MM contributed to the conception and design of the study and wrote the manuscript. All authors contributed to manuscript revision, read, and approved the submitted version.

## Funding

This work was supported by grants from Instituto Nacional del Cáncer (INC-MSAL,II Convocatoria, 2016 and VI Convocatoria, 2020), Fondo Tecnológico Córdoba-MINCyT Cba and Fundación para el Progreso de la Medicina. Programa SECYT-UNC 30920190100043CB. FONCyT-PICT 2018-1789. Proyectos Institucionales de IDTyA de interés regional 2020 de SeCyT-UNC con fondos de SPU. Res. SeCyT-UNC 322/20.

## Acknowledgments

We thank Vanesa Losi and Fernanda Ponce for their technical assistance in immunohistochemistry assays.

## Conflict of interest

The authors declare that the research was conducted in the absence of any commercial or financial relationships that could be construed as a potential conflict of interest.

## Publisher’s note

All claims expressed in this article are solely those of the authors and do not necessarily represent those of their affiliated organizations, or those of the publisher, the editors and the reviewers. Any product that may be evaluated in this article, or claim that may be made by its manufacturer, is not guaranteed or endorsed by the publisher.
